# Economic impact of dengue in Mexico considering reported cases for 2012 to 2016

**DOI:** 10.1371/journal.pntd.0006938

**Published:** 2018-12-14

**Authors:** Adriana Zubieta-Zavala, Malaquias López-Cervantes, Guillermo Salinas-Escudero, Adrian Ramírez-Chávez, José Ramos Castañeda, Sendy Isarel Hernández-Gaytán, Juan Guillermo López Yescas, Luis Durán-Arenas

**Affiliations:** 1 Faculty of Medicine, National Autonomous University of Mexico, Mexico City, Mexico; 2 Center for Economic Studies and Social Health, Children’s Hospital of Mexico Federico Gómez, Ministry of Health, Mexico City, Mexico; 3 Private Practice, Mexico City, Mexico; 4 Departamento de Arbovirus, National Institute of Public Health, Cuernavaca, Mexico; 5 Medical Affairs, Sanofi Pasteur Latin America, Mexico City, Mexico; Brandeis University, UNITED STATES

## Abstract

**Background:**

Given that dengue disease is growing and may progress to dengue hemorrhagic fever (DHF), data on economic cost and disease burden are important. However, data for Mexico are limited.

**Methodology/Principal findings:**

Burden of dengue fever (DF) and DHF in Mexico was assessed using official databases for epidemiological information, disabilities weights from Shepard et al, the reported number of cases and deaths, and costs. Overall costs of dengue were summed from direct medical costs to the health system, cost of dengue to the patient (out-of-pocket expenses [medical and non-medical], indirect costs [loss of earnings, patient and/or caregiver]), and other government expenditures on prevention/surveillance. The first three components, calculated as costs per case by a micro-costing approach (PAATI; program, actions, activities, tasks, inputs), were scaled up to overall cost using epidemiology data from official databases. PAATI was used to calculate cost of vector control and prevention, education, and epidemiological surveillance, based on an expert consensus and normative construction of an ideal scenario.

Disability-adjusted life years (DALYs) for Mexico in 2016 were calculated to be 2283.46 (1.87 per 100,000 inhabitants). Overall economic impact of dengue in Mexico for 2012 was US$144 million, of which US$44 million corresponded to direct medical costs and US$5 million to the costs from the patient’s perspective. The estimated cost of prevention/surveillance was calculated with information provided by federal government to be US$95 million. The overall economic impact of DF and DHF showed an increase in 2013 to US$161 million and a decrease to US$133, US$131 and US$130 million in 2014, 2015 and 2016, respectively.

**Conclusions/Significance:**

The medical and economic impact of dengue were in agreement with other international studies, and highlight the need to include governmental expenditure for prevention/surveillance in overall cost analyses given the high economic impact of these, increasing the necessity to evaluate its effectiveness.

## Author summary

Dengue fever is caused by a flavivirus transmitted predominantly by the mosquito *Aedes aegypti*. Infection causes a broad spectrum of clinical signs and symptoms, from mild disease, such as dengue fever to a life threatening form known as dengue hemorrhagic fever. The disease is widespread in tropical regions. Measures such as vector control can slow the spread of infection, and most countries where the disease is endemic, Mexico included, have programs in place to this end. However, faced with other health issues that also require attention, it is important to quantify the suffering caused by dengue and also its economic costs. In this study, we aimed to produce detailed figures for Mexico to complement and refine those available from international studies. Such information will help guide how the money budgeted for health in dengue is spent.

## Introduction

Dengue fever (DF) is a vector-borne viral infection, the incidence of which has increased and expanded geographically over the past 50 years. The World Health Organization estimates reported a total of 50–100 million infections per year for the period 2010 to 2013 [1]. According to the Global Burden of Disease 2016 Study [2], there was a significant increase in mortality from dengue between 2006 and 2016; from 20,800 deaths (95% uncertainty interval [UI] 6000–26,500) in 2006 to 37,800 (95% UI 10,900–52,700) deaths in 2016, while age-standardized rates increased from 0.3 deaths per 100,000 (95% UI 0.01–0.4) in 2006 to 0.5 (95% UI 0.2–0.7) deaths per 100,000 in 2016. Modeling of the incidence of dengue, accounting for under-reporting of cases, has also showed an increase between 1990 (8.3 million cases [95% uncertainty estimate 3.3 million–17.2 million]) and 2013 (58.4 million cases [95% uncertainty estimate 23.6 million–121.9 million])[3].

Clinical symptoms of DF can lead to a wide range of clinical manifestations; these are usually mild but some patients may be hospitalized, and progress to a more severe and life-threatening form of the disease that requires admission to an intensive care unit (ICU) [4]. The potentially severe consequences of infection, allied with the high prevalence, especially during epidemic years, make for a high burden of disease and high economic cost [5]. Nevertheless, other health issues compete for limited overall resources, so it is important to have reliable figures to quantify as accurately as possible the burden and costs of dengue to enable rational budget allocation.

The economic and disease burden of dengue in Mexico has previously been estimated for the period 2010–2011 by Undurraga et al [6], and was recently updated by Tiga et al [7] who also considered the persistent symptoms of dengue. In addition, Shepard et al [8] estimated the direct and indirect costs of hospitalized and ambulatory dengue episodes from several countries, including Mexico.

Mexico has complex arrangements for healthcare in which two main systems, the Secretariat of Health (SS) and the Mexican Social Security Institute (IMSS), coexist [9]. We have previously reported the estimated direct costs per case for both the health system and for patients, along with the indirect costs per case, using a micro-costing approach known as the program, actions, activities, tasks, inputs (PAATI) method [10]. These costs per case then need to be scaled up to the overall population, and the additional cost of prevention/education programs and other governmental expenses (such as the cost of surveillance) need to be considered to obtain a more complete picture of the overall medical and economic impact of dengue in Mexico.

This article reports epidemiological data extracted from databases provided by the National Center for Disease Control and Prevention (Centro Nacional de Programas Preventivos y Control de Enfermedades, CENAPRECE), the National System for Epidemiological Surveillance (Sistema Nacional de Vigilancia Epidemiológica, SINAVE) and the General Health Directorate. With this information, data from other federal sources on additional costs (such as prevention/education programs and surveillance), and the results of surveys, we report here the estimates for the overall direct medical costs to the health system, costs from the patient perspective, governmental costs of the system, and economic impact of dengue in Mexico for 2012 to 2016.

## Methods

### Epidemiological estimation of the total episodes of dengue

Given that only limited analyses of epidemiological data for dengue have been published for Mexico at the time of analysis, epidemiological data were derived from databases provided by CENAPRECE, SINAVE and the General Health Directorate for 2012–2016. For each of these years, the overall number of cases of DF and DHF and the number of deaths attributed to dengue infection were extracted.

### Burden of dengue

The disease burden was estimated for 2016 by calculating three measures of Years of Life Lost (YLL), Years Lost due to Disability (YLD), and Disability-Adjusted Life-Years (DALYs). The YLL were estimated as the difference between the age of death and the life expectancy corresponding to those who survive at that age at the time of death; life expectancy was based on the average life expectancy reported by INEGI (National Institute of Statistics and Geography) in Mexico [11]. The YLD were calculated as the product of the number of cases that have a certain health status, the duration of that health status and the weight of the disability for that state of health, adjusted to annual values.

To estimate the YLD we considered a systematic review by Shepard et al, whom estimated specifically dengue, not general infections [12]. The disability weight used was 0.032 (0.018–0.044) for DF and 0.036 (0.022–0.050) for DHF, and the average duration days were 11.5 days for DF and 14.2 days for DHF. The expectancy of life (75 years) was considered to be distributed homogeneously for the cases of DF and DHF [11]. The incidence (number of cases) by age was determined by consulting SINAVE [13].

We present a second calculation with all the previous estimations but considering all symptomatic dengue infections with an expansion factor of 5.6 for ambulatory and 2.0 for hospitalized, as described in Undurraga et al [6]. The expansion factor was applied to DF, DHF, and deaths.

### Economic impact of dengue

A multi-method approach was used to calculate the economic impact of dengue. The different elements of the estimation are presented in the following formula:

Economicimpactofdengue=[costpercase]×[totalepisodes]+[costofdenguepreventionandsurveillanceprogram]

Each element of the formula is explained below. All costs were calculated in local currency (Mexican pesos), converted to US Dollars using the exchange rate on September 12, 2012 (1 US$ = 13.03 Mexican pesos) and then adjusted for inflation for each year.

### Cost per case

#### PAATI methodology

The cost per case was calculated using a hierarchical micro-costing approach to quantify direct costs incurred by the healthcare services; PAATI. Considering Gold et al [14], PAATI would be classified as a micro-costing tool as it refers to a direct accounting, with the cost of each of the inputs used in the treatment of a particular patient. However, considering the processing of the information, it is defined as a bottom-up approach, which refers to valuing each patient, or input, individually. Through this method, the cost is assigned to the inputs, which allows the estimation of the cost per procedure. Given its construction and structure, it has also been used as a model to evaluate the interaction of program components. A normative or ideal version of PAATI is based on the program’s documentation, opinion of experts and normative documentation from national and international agencies. For the construction of the normative PAATI model, two basic and interactive sources were used; the latest normative and technical documents at the time of analysis, and the consensus of operative technical program advisors, whom base the ideal terminal design of the program.

**Fig 1:**
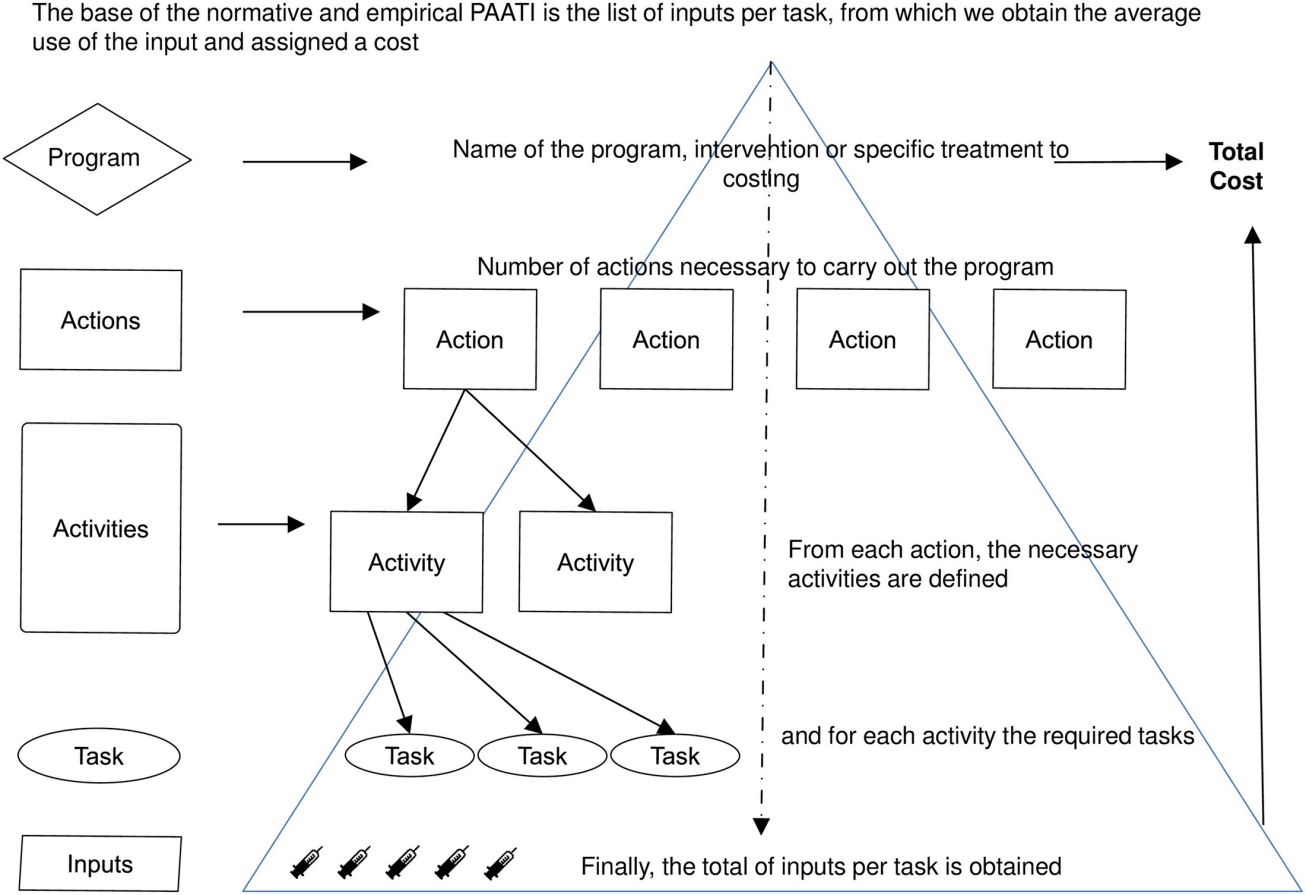
General scheme of PAATI.

Once the model is constructed, the cost of the normative or ideal PAATI is estimated up to the cost of inputs according to the established ideal, in such a way that the sum of all the inputs allows the representation of the cost for the program, ideal but not unreal. Empirical or real PAATI is built by reviewing the application of the program in the field, that is, the normative or ideal PAATI is contrasted with the reality of the operation of the program. Both, ideal or normative, and real or empirical, are built following the same scheme (Fig 1) but accordingly to the established normativity or reality of the operation.

A panel of experts agreed on an ideal protocol for the treatment of a case of DF or DHF in a meeting held on June 5, 2012, the protocol was drafted using a review, and reference to treatment guidelines for Mexico and Latin America [4,15,16,17,18,19], and This protocol was used in an expert group discussion, and the authors of this paper actively participated (Betancourt-Cravioto et al. [20] and see Zubieta-Zavala [10] for further details). The protocol covered diagnosis, case identification, classification and notification, and treatment. The costs assigned to each of the inputs in the protocol were summed to give a cost per case in the ideal or normative scenario (in both the SS and IMSS settings). Subsequently, the real or empirical costs were calculated by determining the inputs actually taken from a field survey of data from 1293 patients from 32 hospitals and 32 ambulatory centers in 16 Mexican states with endemic DF.

#### Cost per case calculation

The cost per case used for this estimation was calculated by summing three categories; for more details about ideal and real costs see the previous article [10]: (1) direct medical costs incurred by healthcare units (ambulatory, hospital or ICU); (2) costs of dengue from the patient’s perspective (direct medical costs not covered by the public healthcare services and direct non- medical costs, eg travel expenses); (3) indirect costs (to the patient and their family) resulting from loss of productivity. Indirect costs from the patient’s perspective were calculated based on information derived from interviews with patients. For these data, the methodology took into account costs reported by the patients, and these were used to calculate the average cost per case, as well as confidence intervals. A bootstrap analysis was performed to assess variability.

It is important to clarify that cost of each activity calculated in the direct medical cost incurred by healthcare units was calculated using the average use reported per input (ie resource or cost type). Each separate type of cost incurred was looked at, which allows control of the variability for each component of the healthcare process, rather than using other methodologies where the average overall cost per patient is calculated and then assigned to each activity.

### Adjusting reported cases

Previous studies in Mexico have reported a high level of under-reported and under-notification of cases [6,21,22]. Initially not all symptomatic patients were considered, only cases reported to the system. Nevertheless, in order to allow for comparability with other studies, a sensitivity analysis was conducted, where the base case is with no factor expansion, followed by two different scenarios with two expansion factors taken from the study by Undurraga et al [6]; the dengue patients who visited a health facility (3.7 ambulatory and 1.4 hospitalized), and considering all symptomatic dengue infections (5.6 ambulatory and 2.0 hospitalized). Finally the calculation follows the same assumptions as the “Economic Impact of dengue” section. It is important to clarify that the ambulatory expansion factor by Undurraga et al is the only one applicable to this cost data. In that sense Martinez-Vega, et al reported a general 33.3% under-reporting and 68.2% under-notified [22]; while Sarti et al reported an 8.4-fold local expansion factor [21]. In both studies we do not know the differences by healthcare setting, and so these calculations were not included in our study.

Although comparisons with other countries may be illustrative, firm conclusions cannot be drawn given the differences in economic development, population size, and healthcare systems, as well as the methodology used for the estimates.

### Cost of dengue prevention and surveillance program

**Fig 2:**
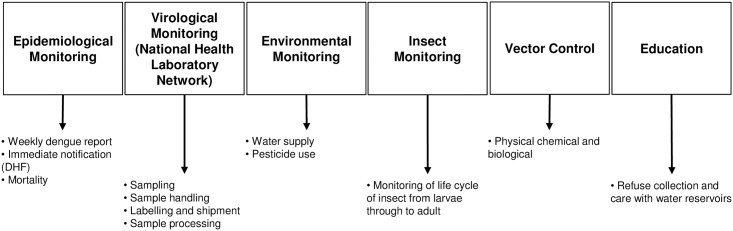
Overview of epidemiological PAATI.

Estimation of the economic cost of dengue should also include the cost of vector control and prevention, education, and epidemiological surveillance. As for cost per case, costs for dengue prevention and surveillance activities were also calculated using the PAATI approach, following the same scheme previously mentioned (Fig 1). An ideal protocol was prepared using a literature review and guidelines from the Mexican program [16,17], and then the support and consensus of federal technical advisor users of the program who also participated in the expert group discussion sponsored by the Ministry of Health was sought (see Betancourt-Cravioto et al. [20]). Six major activities were identified (Fig 2)(epidemiological surveillance, virological surveillance, environmental surveillance, insect surveillance, vector control and education).

#### DHF, dengue hemorrhagic fever; PAATI, programs, actions, activities, tasks, and inputs

Three essential criteria of the normative or ideal PAATI were included: (1) structural simplification, delimiting the substantial components of the system, in a structure consistent with the operative reality, minimizing its complexity; (2) operational organization, to outline the progressive order of application of each component, from a global scale to the precise or specific detail that integrate the tasks and necessary and sufficient inputs to achieve the overall objective or purpose; and (3) schematic structure of inputs and necessary costs. Based on these criteria, a simple and orderly outline of the detail of the inputs and costs necessary from a normative perspective was constructed, as a component of the program for the prevention and control of the transmission of dengue.

Once the ideal or normative protocol had been drafted, the input costs were estimated for every task. The sum of all inputs was calculated for blocks of 10,000 individuals, grouped in basic geostatistical areas. Unlike the PAATI for cost per case, this PAATI is not applied to individuals because the program performs its activities for populations. The overall cost was then calculated by summing the contribution of each block of 10,000 inhabitants at risk of infection (i.e., in states where dengue is endemic). Finally, the “epidemiological ideal PAATI” was estimated with the information reported at the federal level based on the report of budgetary items and source of general funding.

In the real scenario or empirical PAATI, costs were associated with current practice (which we denote ‘epidemiological real PAATI’). The person responsible for the dengue program in each of the states in the study was contacted and provided a questionnaire to directly determine the expenses incurred in the control program. The information given was limited, and it was not possible to determine the ‘epidemiological real cost’. Although ‘real’ estimates of surveillance and prevention costs would have been a useful contribution, the variability between states due to the lack of information prevents us presenting the comparison. Consequently the current estimation is presented with the “epidemiological ideal PAATI”

### Economic impact of dengue

The calculation of the economic impact of dengue was based on three assumptions: (1) the direct costs were considered by the IMSS clinical real PAATI; although, we have data from a previous study for IMSS and SS, we consider costing for the system with the best quality in following the protocol, in order to have the most accurate estimation, and so IMSS alone was used; (2) as not all patients have out-of-pocket expenses, the costs from a patient perspective were estimated to be a proportion of the population, taken directly from previous data: 38.5%, 4% and 5% (proportion of patients with expenses) of the total cost per case for hospital cases, outpatients and patients in the ICU, respectively; and (3) the estimated cost of the dengue prevention and control program is the ideal epidemiological PAATI for the population of 25 endemic states.

### Other economic impacts of dengue

Despite attempting to estimate cost of dengue on the health system, patients and government, it was not possible to determine all types of costing from these sources. For example, persistent cases or sequelae following infection were not considered [7,23]. Data limitations prevented the quantification of the impact of tourism, or the impact of dengue on tourism revenues. Although, dengue cost might include other hidden items [24]. The study was delimitated to the points made in the methodology section.

## Results

### Epidemiology

Consultation of SINAVE, run by the General Directorate of Epidemiology, revealed three peaks in DF during the period studied (from 2012 through 2016, the most recent update to the database). The peak is presented in 2013 with 105,973 DF and 19,822 DHF cases (Table 1). The highest number of deaths was reported in the same year and the case fatality rate for DHF have increased in this period, with the highest rate of 1.70% reported in 2015.

**Table 1:** Dengue fever, dengue hemorrhagic fever and mortality in Mexico, 2012–2016.

Year	Cases of dengue fever	Cases of dengue hemorrhagic fever	Deaths	Case fatality rate (%)^a^
2012	65,892	18,720	162	0.86
2013	105,973	19,822	187	0.94
2014	46,092	8856	93	1.0
2015	61,710	5626	96	1.70
2016	41,907	3717	63	1.69

^a^Relative to cases of dengue hemorrhagic fever

### DALYs associated with dengue in Mexico

**Fig 3:**
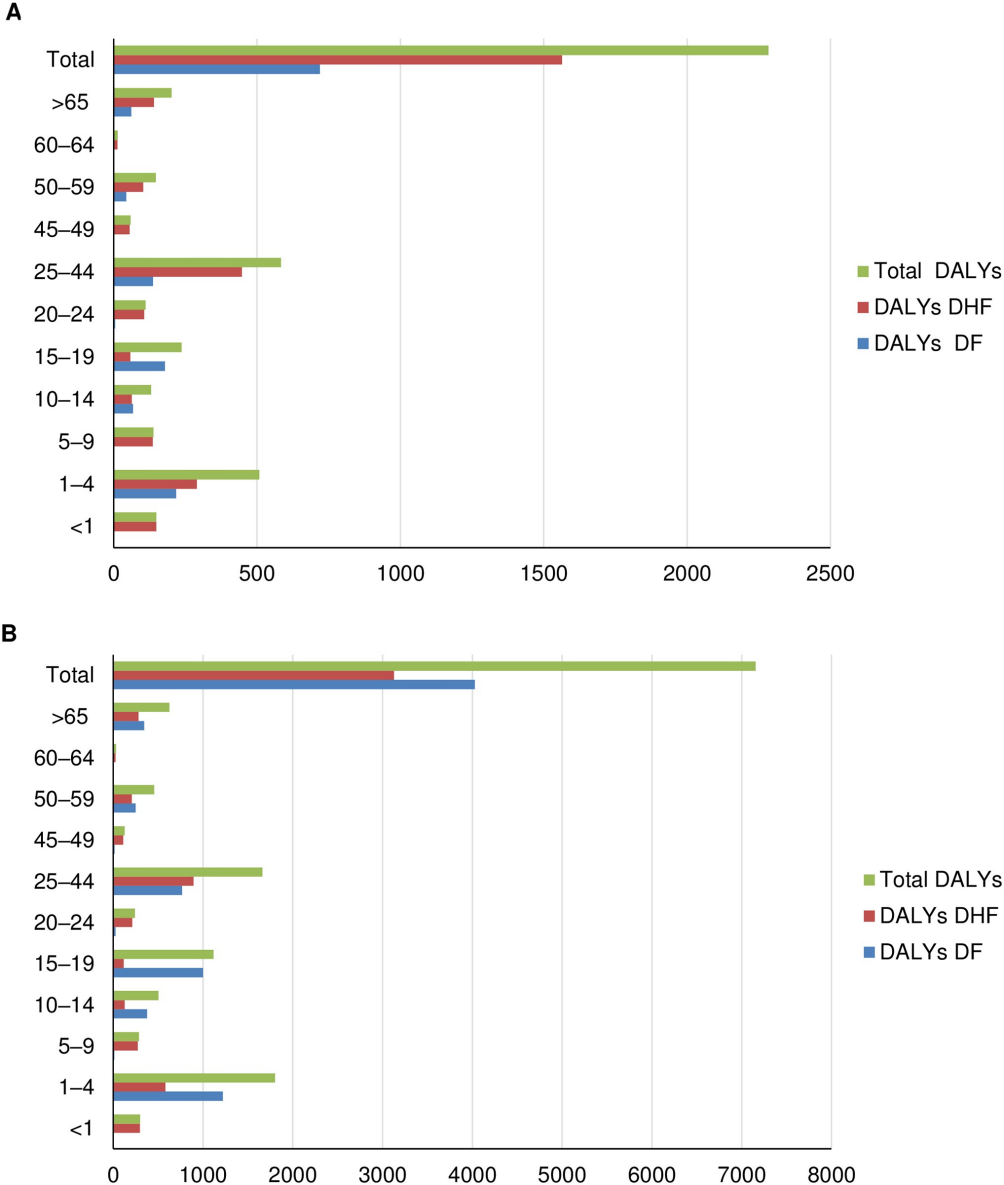
Comparison DALYs for DF, DHF and total in 2016 by age group without (A) and with (B) expansion factors.

**Fig 4:**
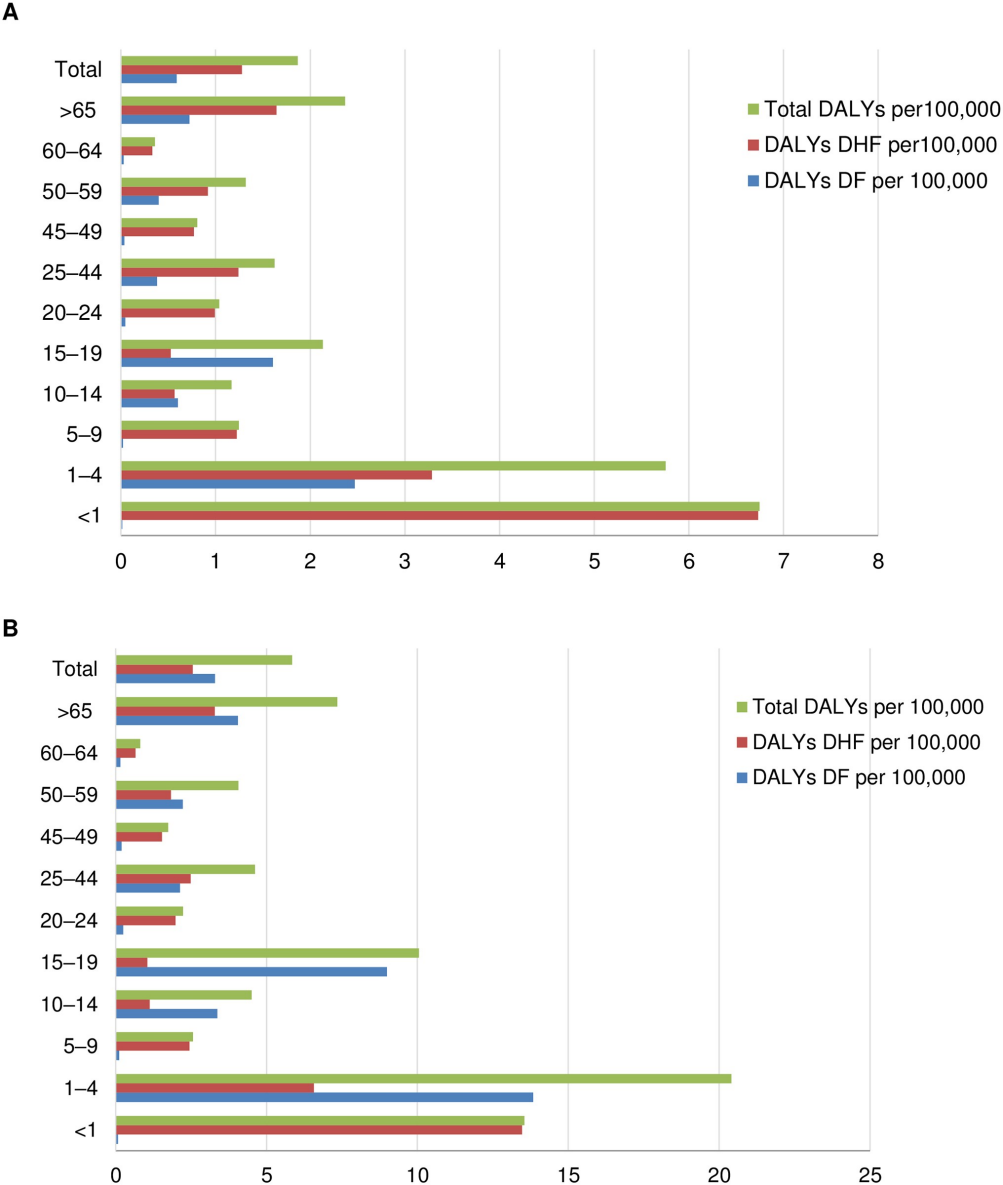
Comparison DALYs per 100,000 inhabitants for DF, DHF and total in 2016 by age group without (A) and with (B) expansion factors.

Using the above epidemiological data, the DALYs for Mexico in 2016 were calculated to be 2283.46 which correspond to 1.87 DALYs per 100,000 inhabitants (Table 2). With the application of expansion factors for all symptomatic cases (Table 3), we observed that DALYs were 7156.22 which correspond to 5.85 DALYs per 100,000 inhabitants. As shown in Figs 3 and 4, this burden fell largely on the younger age groups (1–4 years). The main driver corresponded to the ‘early death’ component (YLL) as the ‘living with disability’ component (YLD) contributed less than the overall DALYs (Tables 2 and 3). On application of the expansion factor, the change is proportional in each age group.

**Table 2:** Burden of disease for DF and DHF in 2016 by age group.

Age group	Population	Deaths DF	Deaths DHF	YLL DF	YLL DHF	YLL Dengue	YLD DF	YLD DHF	YLD Dengue	DALY Dengue	YLL per 100,000	YLD per 100,000	DALYS per 100,000
<1	2,213,347	0	2	0	149	149	0.31	0.05	0.36	149.36	6.73	0.02	6.75
1–4	8,833,106	3	4	217.5	290	507.5	0.77	0.15	0.93	508.43	5.75	0.01	5.76
5–9	11,132,907	0	2	0	136	136	2.33	0.32	2.65	138.65	1.22	0.02	1.25
10–14	11,195,387	**1**	**1**	63	63	126	4.47	0.51	4.97	130.97	1.13	0.04	1.17
15–19	11,128,211	3	1	174	58	232	4.73	0.68	5.41	237.41	2.08	0.05	2.13
20–24	10,747,976	0	2	0	106	106	4.90	0.62	5.52	111.52	0.99	0.05	1.04
25–44	36,000,200	3	11	121.5	445.5	567	15.56	1.57	17.13	584.13	1.57	0.05	1.62
45–49	7,305,568	0	2	0	56	56	2.63	0.29	2.92	58.92	0.77	0.04	0.81
50–59	11,196,064	2	5	41	102.5	143.5	3.61	0.46	4.07	147.57	1.28	0.04	1.32
60–64	3,984,821	0	1	0	13	13	1.11	0.17	1.28	14.28	0.33	0.03	0.36
>65	8,535,897	6	14	60	140	200	1.82	0.40	2.22	202.22	2.34	0.03	2.37
**Total**	**122,273,484**	**18**	**45**	**677**	**1559**	**2236**	**42.25**	**5.21**	**47.46**	**2283.46**	**1.83**	**0.04**	**1.87**

DALY, disability adjusted life years; DHF, dengue hemorrhagic fever; DF, dengue fever; YLD, years lost due to disability; YLL, years of life lost.

**Table 3:** Burden of disease for DF and DHF in 2016 by age group with expansion factors.

Age group	Population	Deaths DF	Deaths DHF	YLL DF	YLL DHF	Total YLL dengue	YLD DF	YLD DHF	Total YLD dengue	Total DALY dengue	YLL per 100,000	YLD per 100,000	DALYs per 100,000
<1	2,213,347	0	4	0	298	298	1.74	0.10	1.85	299.85	13.46	0.08	13.55
1–4	8,833,106	16.8	8	1218	580	1798	4.32	0.31	4.63	1802.63	20.36	0.05	20.41
5–9	11,132,907	0	4	0	272	272	13.06	0.63	13.70	285.70	2.44	0.12	2.57
10–14	11,195,387	5.6	2	352.8	126	478.8	25.02	1.01	26.03	504.83	4.28	0.23	4.51
15–19	11,128,211	16.8	2	974.4	116	1090.4	26.46	1.36	27.82	1118.22	9.80	0.25	10.05
20–24	10,747,976	0	4	0	212	212	27.46	1.24	28.69	240.69	1.97	0.27	2.24
25–44	36,000,200	16.8	22	680.4	891	1571.4	87.16	3.13	90.29	1661.69	4.36	0.25	4.62
45–49	7,305,568	0	4	0	112	112	14.73	0.57	15.30	127.30	1.53	0.21	1.74
50–59	11,196,064	11.2	10	229.6	205	434.6	20.21	0.92	21.12	455.72	3.88	0.19	4.07
60–64	3,984,821	0	2	0	26	26	6.23	0.33	6.57	32.57	0.65	0.16	0.82
>65	8,535,897	33.6	28	336	280	616	10.21	0.80	11.01	627.01	7.22	0.13	7.35
**Total**	**122,273,484**	**100.8**	**90**	**3791.2**	**3118**	**6909.2**	**236.61**	**10.41**	**247.02**	**7156.22**	**5.65**	**0.20**	**5.85**

DALY, disability adjusted life years; DHF, dengue hemorrhagic fever; DF, dengue fever; YLD, years lost due to disability; YLL, years of life lost.

### Economic impact of dengue

Table 4 provides the total cost by year and number of cases for direct medical costs and costs from the patient’s perspective, for the years 2012–2016, by care setting and according to the assumptions on the direct medical and patient costs (see Methods section) and the cost per case estimated previously [10].

**Table 4:** Total cost (direct medical costs and the cost to the patient and caregiver) of DF by year and care setting.

Year	Care setting	Direct Medical Costs to the Healthcare System* US$ Average cost	Costs of dengue from the patient’s perspective (direct medical costs + direct nonmedical costs + productivity loss) US$ Average cost (95%CI)^†^	Totals US$ Average cost
**2012**	Outpatients	$6,064,041	$103,767 ($45,597–$163,834)	$6,167,807
	Hospitalized patients	$29,249,167	$4,599,711 ($2,229,167–$8,576,544)	$33,848,878
	Patients in ICU	$8,774,710	$228,898 ($148,052–$373,229)	$9,003,608
	Patients managed in all three settings	$44,087,918	$4,932,375 ($2,422,817–$9,113,607)	**$49,020,293**
**2013**	Outpatients	$10,346,634	$177,050 ($77,799–$279,538)	$10,523,684
	Hospitalized patients	$32,857,126	$5,167,097 ($2,504,141–$9,634,482)	$38,024,223
	Patients in ICU	$9,857,093	$257,133 ($166,314–$419,268)	$10,114,226
	Patients managed in all three settings	$53,060,853	$5,601,279	**$58,662,133**
**2014**	Outpatients	$4,635,180	$79,316 ($34,853–$125,230)	$4,714,497
	Hospitalized patients	$15,120,179	$2,377,793 ($1,152,355–$4,433,592)	$17,497,972
	Patients in ICU	$4,536,033	$118,327 ($76,534–$192,939)	$4,654,360
	Patients managed in all three settings	$24,291,393	$2,575,436	**$26,866,829**
**2015**	Outpatients	$6,391,957	$109,378 ($48,063–$172,693)	$6,501,335
	Hospitalized patients	$9,893,644	$1,555,870 ($754,024–$2,901,049)	$11,449,514
	Patients in ICU	$2,968,080	$77,426 ($50,079–$126,246)	$3,045,505
	Patients managed in all three settings	$19,253,681	$1,742,674	**$20,996,355**
**2016**	Outpatients	$4,470,974	$76,506 ($33,619–$120,794)	$4,547,480
	Hospitalized patients	$38,375,956	$1,058,774 ($513,116–$1,974,173)	$39,434,730
	Patients in ICU	$2,019,787	$52,688 ($34,079–$85,911)	$2,072,476
	Patients managed in all three settings	$44,866,717	$1,187,969	**$46,054,686**

* PAATI methodology;

^†^costs reported by the patients, a bootstrap analysis was performed to assess variability

This expense (shown in Table 4) represents the cost to the system and the cost to the patient and caregiver.

Governmental costs related to dengue were prevention, surveillance and control programs, and total cost of the ideal epidemiological PAATI were estimated to be US$11,766.52 per 10,000 inhabitants. As shown in Table 5, vector control was the biggest cost driver, accounting for approximately 40% of the overall nonmedical governmental costs, followed by epidemiological surveillance, which accounted for approximately 25%.

**Table 5:** Breakdown of dengue prevention, surveillance and control program costs in an ideal scenario using the PAATI methodology.

Action	Partial cost per 10,000 inhabitants
Epidemiological surveillance	$3,220.98
Virological surveillance	$1,320.00
Environmental surveillance	$813.24
Insect surveillance	$502.68
Vector control	$4,847.78
Education	$1,061.84
**Total cost per 10,000 inhabitants**	**$11,766.52**

Finally, Table 6 shows the overall economic impact of dengue infection for 2012–2016, according to the assumptions mentioned previously, and shows the differences between our results with and without the expansion factors between 2012 and 2016.

**Table 6:** Total economic impact of dengue infection 2012–2016, considering 25 endemic states in Mexico and differences between expansion factors.

Year	Applied Factor	Direct Medical Costs to the Healthcare System* (US$ Average use cost)	Total cost of dengue from the patient’s perspective(direct medical costs + direct nonmedical costs + productivity loss) (US$ Average cost, 95%CI)	Total Program cost in 25 endemic states* (US$)	Total economic Impact (US$)
**2012**	**Without factor**	$44,087,916	$4,932,375 ($2,422,817–$9,113,607)	$95,441,720	$144,462,012
	**Health Facility**	$75,670,378	$7,143,989 ($3,496,817–$13,135,868)		$178,256,087
	**All SYM**	$110,006,382	$10,238,311 ($5,009,783–$18,817,016)		$215,686,412
**2013**^†^	**Without factor**	$53,060,853	$5,601,279 ($2,748,255–$10,333,288)	$102,454,898	$161,117,031
	**Health Facility**	$98,082,454	$8,249,005 ($4,026,495–$15,109,541)		$208,786,358
	**All SYM**	$143,369,590	$11,839,938 ($5,776,587–$21,672,913)		$257,664,426
**2014**^†^	**Without factor**	$24,291,393	$2,575,436 ($1,263,742–$4,751,761)	$106,742,637	$133,609,466
	**Health Facility**	$44,668,865	$3,788,038 ($1,849,402–$6,940,494)		$155,199,540
	**All SYM**	$65,269,435	$5,436,411 ($2,652,956–$9,954,349)		$177,448,483
**2015**^†^	**Without factor**	$19,253,681	$1,742,674 ($852,167–$3,199,989)	$110,432,820	$131,429,174
	**Health Facility**	$41,656,655	$2,691,312 ($1,303,578–$4,877,179)		$154,780,787
	**All SYM**	$55,582,249	$3,879,108 ($1,877,360–$7,021,673)		$169,739,325
**2016**^†^	**Without factor**	$13,223,416	$1,187,969 ($580,813–$2,180,877)	$115,794,366	$130,205,751
	**Health Facility**	$24,839,701	$1,839,121 ($890,462–$3,331,053)		$146,429,509
	**All SYM**	$38,502,762	$2,651,361 ($1,282,654–$4,796,611)		$156,843,113

* PAATY methodology;

^**†**^years adjusted by inflation

Health facility: Patients who visited a health facility, expansion factor 3.7 ambulatory and 1.4 hospitalized; All SYM: All symptomatic dengue infections, expansion factor 5.6 ambulatory and 2.0 hospitalized.

The overall economic impact of dengue infection (DF and DHF) for 2012 to 2016 shows how the cost is proportional to the number of cases obviously, but the real impact on cost is the prevention and control program. It is important to mention that the cost of the prevention program was not calculated for each year because the method essentially involves identifying the tasks and inputs from an ideal protocol and then assigning a unit cost to each. Therefore, there was no estimated average for the program. It is also important to emphasize that this estimation is not a projection. It was made using the reported cases to the system per year in Mexico and the cost calculation previously explained. The economic impact of dengue was presented by year from 2012 (reference year) to 2016, the most recent year for which epidemiological information was available, with adjustment for inflation by year. As shown in Tables 1 and 6, 2012 had 65,892 DF cases and an economic impact of US$144 million. The increment in the cost of the program is not proportional to the cases because execution of the program does not depend on the number of cases. Although 2016 had fewer cases than the others, the overall cost is only slightly affected.

Additionally, expansion factors for patients with dengue who visited a health facility and all symptomatic dengue infections were used in order to compare these results with the previous studies [6]. Although comparisons with other countries may be illustrative, firm conclusions cannot be drawn given the differences in economic development, population size, and healthcare systems, as well as the methodology used for the estimates.

## Discussion

In the present analysis, considering only the reported cases, the cost of dengue infection in Mexico in 2012 was estimated to be US$144 million. In other studies of the disease in Mexico, Undurranga et al [6] estimated a cost of US$149 million (95% CI: $136–$231) in 2011 and an average of US$170 million (95% CI: $151–$292) for 2010 and 2011. Tiga et al [7] reported an estimated average annual cost of US$192 million (95% CI: $171– $325) in 2012; however, it has to be considered that these estimates include persistent symptoms, and therefore must be compared to our outcomes with caution. The number of cases reported in 2011 was lower than in this study (2012–2016). To investigate the impact this lower incidence might have, estimated costs for 2011 using the same assumptions, with adjustment for inflation were also calculated. The cost obtained for 2011 was US$103, showing that these estimates are lower than other published estimates for each year.

First, this must be considered as a consequence of using the PAATI methodology which, as a micro-costing methodology, starts with a detailed inventory and measurement of inputs that detects small differences in cost. It is more accurate than a macro-costing or a top-down approach, which are the typical analysis methods for aggregate data [25,26]. It is important to mention that only the clinical PAATI had a top-down approach, the epidemiology data used a bottom-up approach, although both are micro-costing. Second, PAATI was calculated using the average use reported per input (ie resource or cost type). Each separate type of cost incurred was examined, which allows control of the variability for each component of the healthcare process, rather than using other methodologies where the average overall cost per patient is calculated and then assigned to each activity. Third, the expansion factor also has an impact; when these finding are compared with the adjustments in Table 6 it is clear that the estimation is getting closer to the other published studies. Although the expansion factor was added to enable comparison with other studies, the raw number of cases reported to the surveillance system in the 25 endemic states were used initially; it is important to clarify that it was not the aim to pronounce this as the best estimation.

As mentioned previously, the cost of the government programs account for the majority of the overall cost and the actual number of cases has a limited impact. Nevertheless, the direct costs, derived from the number of cases, may be an underestimate of the actual cost given that DF is thought to be subject to considerable under-reporting. During the latest years, the number of cases of dengue have increased considerably, and several studies have shown number of cases to be underestimated. For example, a recent review of the global distribution and burden of dengue concluded that total infection was more than three times the dengue burden estimate of the World Health Organization [27]. In the study by Shepard and colleagues [28], an expansion factor of 15 for DF was recommended for Mexico the recommended expansion factor for DHF was 2.3. The results of a prospective cohort study, whose main aim was to investigate peridomestic infection as a determinant of dengue transmission, supported the expansion factors used by Shepard and colleagues on crosschecking the infections detected in their study with official databases [29]. In a recent study by Sarti et al, expansion factors indicated significant underreporting, and the authors reported that their use should be interpreted with caution [21]. Another study in Puerto Rico estimated the degree of underreporting of dengue cases by building a model using data from two different surveillance systems; the estimated rates of between 2.1 (for inpatients) to 7.8 (for outpatients) per 1,000 population compare with reported rates of 0.4 for outpatients and 0.1 for inpatients per 1,000 population [30].

In recent years, other countries have evaluated the economic impact of dengue. Colombia estimated a financial cost of US$167.8 million for 2010, US$129.9 million for 2011, and US$131.7 million for 2012 [31]. In Brazil, the annual total cost for dengue was estimated to be US$164 million (90% CI: $123–$205) from the public payer perspective and increased to US$ 447 million (90% CI: $335–$559) with adjustment for underreporting [32]. For the societal perspective which includes weight by public and private sector, the estimated cost was US$ 468 million (90% CL: 349–590) and US$ 1,212 million (90% CL: 904–1,526), adjusting for under-reporting [32].

Finally, the overall cost of dengue presented in this study does not include preventive measures taken by households to limit transmission (for example, use of insecticide and bed nets [33]). Clearly, this component may be non-negligible, but it would be extremely difficult to provide a reliable estimate for a number of reasons. First, if a family buys insecticide, for example, it would be difficult to determine what proportion of this expense was specific to the mosquitos that transmit dengue. Second, any survey would be subject to considerable recall bias, not to mention the problems with obtaining a representative sample.

The DALYs calculated for dengue in Mexico were 2283.46, which corresponds to 1.87 per 100,000 inhabitants or 18.67 DALYs per millon. Comparing with the global burden of dengue [3], with fatal and non-fatal outcomes together, dengue was responsible for 1.14 million (0.73 million–1.98 million) DALYs in 2013, which appear to be higher in Mexico. We should consider between these estimations that ongoing factors, such as increased urbanization [34] and climate change [35], have been associated with increased incidence. It seems likely that the number of cases in peak years will continue to increase if preventive measures are not taken. In addition, the DALYs are calculated using only mortality and disability; disruption of healthcare services, productivity losses and broader economic impacts also characterize the burden of dengue illness and are not taken into account in DALYs.

It is also illustrative to compare the burden of dengue obtained in this study with figures reported for other studies, which tend to report a markedly higher burden. For example, a study from before 2000 in Puerto Rico reported a burden of dengue of 658 DALYs per million inhabitants [36]. Another study in Thailand reported a value of 427 DALYs per million inhabitants [37], while a study of several southeast Asian countries reported 372 DALYs per million inhabitants [38]. In Mexico, Undurraga [6] reported the total disease burden for the adjusted average of dengue episodes was 83.5 and 46.7 DALYs per million inhabitants in 2010 and 2011 respectively. All those studies used expansion factors in their calculations. In principle, this could explain the difference between the figure found in this study and the other studies. It is of note, however, that a major component of the overall DALYs was premature death, accounting for more than three-quarters of the overall figure. As most deaths due to dengue are likely to occur in a hospital setting, with attention by specialist professionals, and given the more stringent reporting requirements for deaths, it is hard to imagine that the underreporting rate is high, although this point has been contended [39]. Nevertheless, given the discussion we decided to calculated DALYs with expansion factors and the value was incremented to 58.5 per million. Although we used the expansion factor even in the deaths, the DALYs are still higher in the studies mentioned above.

The study is subject to a number of limitations. IMSS has better data as well as more complete and expensive health care delivery services, so we accept that we could overestimate the cost of the entire system, although; we used costs and not charges, given that the system is public and IMSS do not add a mark up to its costs. In the real PAATI we used current costs as reported by IMSS. At the same time since the system costs are over represented the magnitude of DF is not diminished.

The impact of underreporting on both DALYs and economic cost is likely to mean that the costs and burden of dengue presented here are underestimates. This study used the raw number of cases to provide the base estimate of the real costs when using a PAATI methodology. Nevertheless, when these results are compared using the expansion factor considering the cases of DF not detected by the national surveillance system (3.7 ambulatory and 1.4 hospitalized) a 20% increase is seen, still within the ranges presented in other studies. In addition, as has been discussed previously with the detailed data on costs per case [10], the survey used to derive the real costs is subject to possible sampling bias and incomplete data. However, to compensate, this study has a large sample size which provides more confidence in the stability of the data. It is therefore reassuring that the costs estimated using this micro-costing approach are generally in line with previously published estimates using different methodology. Finally, given the low responses by those responsible for the dengue program in each of the states, it was not possible to present a “real epidemiological PAATI”, and realistic estimates of surveillance and prevention costs would have been a useful contribution. The ideal dengue program will have to be evaluated in future research, with estimates for real costs of the program.

In conclusion, the present study of dengue infection in Mexico shows that the economic impact of dengue is considerable, in broad agreement with other international studies. In particular, the results highlight the need to include not only direct medical and non-medical costs but also the costs linked to surveillance, vector control and prevention in any overall analysis of dengue infection. Given the high economic impact of the prevention, surveillance and control program, their effectiveness needs to be assessed; it cannot be assumed that the lower response in our study is a lack in the program but should be trigger point for research, in the same way for other alternatives, such as vaccination, should also be considered in the next evaluation. However, it is important to remember that many aspects of this program also impact other diseases transmitted by the same vector. Another factor that could be considered in future studies is the geographic distribution of dengue in Mexico. Some regions are highly endemic while others have limited dengue transmission; a review of an endemic sub-region would further inform a more tailored approach in these regions. Although the burden of dengue compared with other infectious diseases may appear limited, the epidemiological trends of mortality, particularly during epidemics, the main driver in the economic impact of this disease, do not leave room for complacency.
